# Intentional Replantation as a Viable Option for Crown-Root Fracture in Maxillary Central Incisor: A Case Report

**DOI:** 10.7759/cureus.57737

**Published:** 2024-04-06

**Authors:** Monika Khubchandani, Ramakrishna Yeluri, Neha Pankey, Meenal Pande

**Affiliations:** 1 Pediatric and Preventive Dentistry, Sharad Pawar Dental College and Hospital, Datta Meghe Institute of Higher Education and Research, Wardha, IND

**Keywords:** prognosis, trauma, intentional replantation, fracture line, crown-root fracture

## Abstract

Crown-root fractures are often challenging to treat and have a poor prognosis. The present case explains the successful management of a vertically fractured tooth treated by intentional replantation in a 12-year-old child. The patient underwent a successful 12-month follow-up, which included a mobility test and measurement of the gingival sulcus depth. Additionally, a radiological assessment was performed to evaluate the root resorption, the integrity of the alveolar cortex, and the periodontal space. We suggest that intentional replantation may be an effective therapeutic approach for the treatment of cases of vertical crown-root fractures.

## Introduction

Anterior teeth are more prone to fracture due to trauma and primarily affect children and adolescents. Most dental injuries involve a single tooth, the maxillary central incisors being the most frequently affected teeth with a reported prevalence of 8.1% [1]. A vertical crown-root fracture (VRF) is a longitudinal (axial) fracture involving the enamel, dentin, and cementum and extends below the gingival margin [2]. As per the reported literature, the incidence of crown-root fracture is 2% in the primary dentition and 5% affecting the permanent dentition usually caused by direct trauma [3]. VRFs are difficult to treat, and the prognosis is dependent on the tooth in question and the extent of the fracture line. Extraction is often the treatment of choice when the fracture line follows the long axis of the tooth. However, when selecting cases carefully, even in cases of vertical fracture, the ease of treatment and prognosis improves [4]. As the alveolar bone resorbs quickly after extraction, every effort should be made to preserve the tooth. Intentional replantation is thought to be considered a viable mode of treatment to preserve the natural dentition. This article presents the management of a tooth in a 12-year-old male child with VRF utilizing intentional replantation as a viable treatment option.

## Case presentation

**Figure 1:**
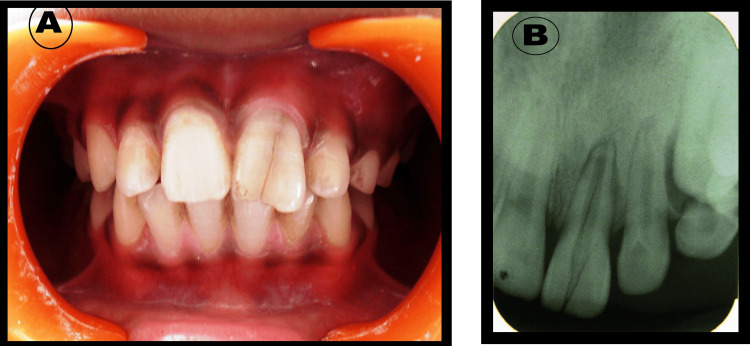
a) Maxillary left central incisor presented with vertical crown fracture. b) IOPA showing two longitudinal vertical fracture lines extending from the crown to the root portion of 21 IOPA: intraoral periapical

A 12-year-old male patient with no significant medical history presented to the Department of Pediatric and Preventive Dentistry with a complaint of a broken upper anterior tooth because of trauma since last year. Intraoral examination revealed a fracture line on the labial side of the crown portion of the maxillary left central incisor (21) (Figure 1A). No tenderness on percussion was observed, and the mobility was within normal limits. The periodontal examination showed normal sulcular depth and normal gingiva. Electric pulp vitality tests revealed that the tooth was non-vital. Intraoral periapical (IOPA) X-ray was performed as a diagnostic measure. The IOPA revealed radiolucent longitudinal vertical fracture lines extending from the crown to the root portion of the maxillary left central incisor (21) (Figure 1B).

**Figure 2:**
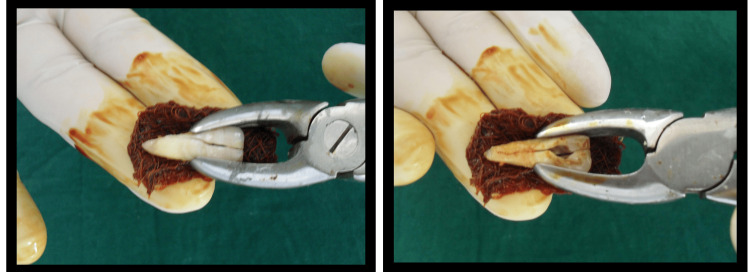
Intentionally extracted maxillary left central incisor showing the fracture lines

Also, widening of the periodontal space was observed along the root surface of 21. Based on clinical history and radiographic examination, the case was diagnosed as VRF along with pulp necrosis in relation to 21. Intentional replantation of the vertically fractured tooth was considered as a possible treatment option. Informed consent was obtained from the patient as well as the parent. Under local anaesthesia, 21 was intentionally extracted. The extracted tooth showed longitudinal incomplete vertical fracture lines at both labial and palatal aspects extending through the cemento-enamel junction (Figure 2).

**Figure 3:**
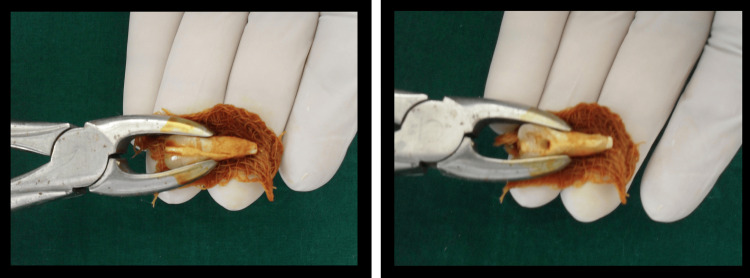
Sealed fracture lines with glass ionomer luting cement, along with sealed root apex

The tooth root was wrapped in a sterile gauze soaked in Betadine solution as a precautionary measure to prevent drying of the root surface. A lingual access cavity was prepared and working length was established with a size 15-k file. The root canal was instrumented and irrigated with sodium hypochlorite, hydrogen peroxide, and normal saline. The buccal and palatal fracture lines were gently air-dried, and both separated fragments were sealed with glass ionomer luting cement (Ketac™ Cem radiopaque, 3M ESPE AG, Seefeld, Germany) (Figure 3). 

**Figure 4:**
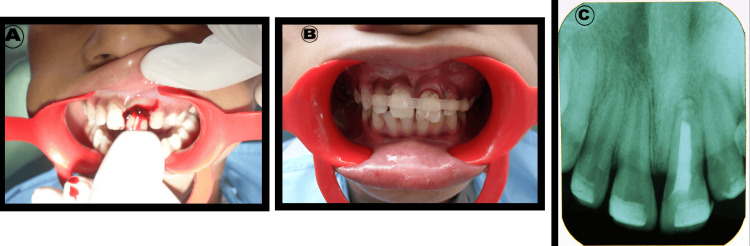
Replantation of maxillary left central incisor followed by stabilization with Ribbond splint

The root apex was sealed with glass ionomer cement (Ketac™ Molar Easymix, 3M ESPE AG, Seefeld, Germany) and the root canal was filled with Pulpdent paste (Pulpdent Corp., Brookline, MA, USA) and finally the access cavity was sealed with glass ionomer cement. The tooth was then replanted into its alveolus and splinted using a polyethylene fiber system (Ribbond, Inc., Seattle, WA, USA) (Figure 4A, 4B). The accurate repositioning of the tooth was confirmed radiographically (Figure 4C).

**Figure 5:**
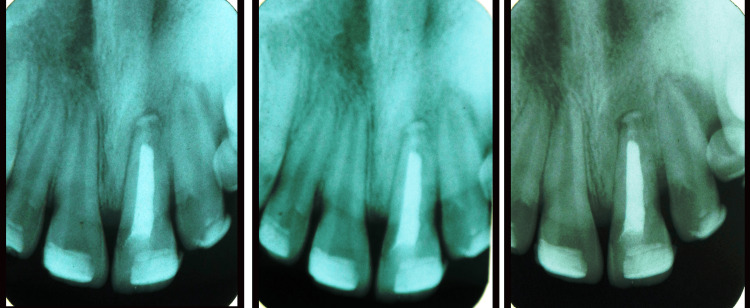
Post-operative IOPA (two, three, and four weeks follow-up) showing partial obliteration of the periodontal space IOPA: intraoral periapical

The entire procedure was performed in approximately nine minutes, and post-operatively, the patient was explained about the importance of plaque control using mechanical and chemical methods. The post-operative period was uneventful, and the patient was followed up at weekly intervals. At the end of four weeks, healing was good and no mobility was observed (Figure 5).

**Figure 6:**
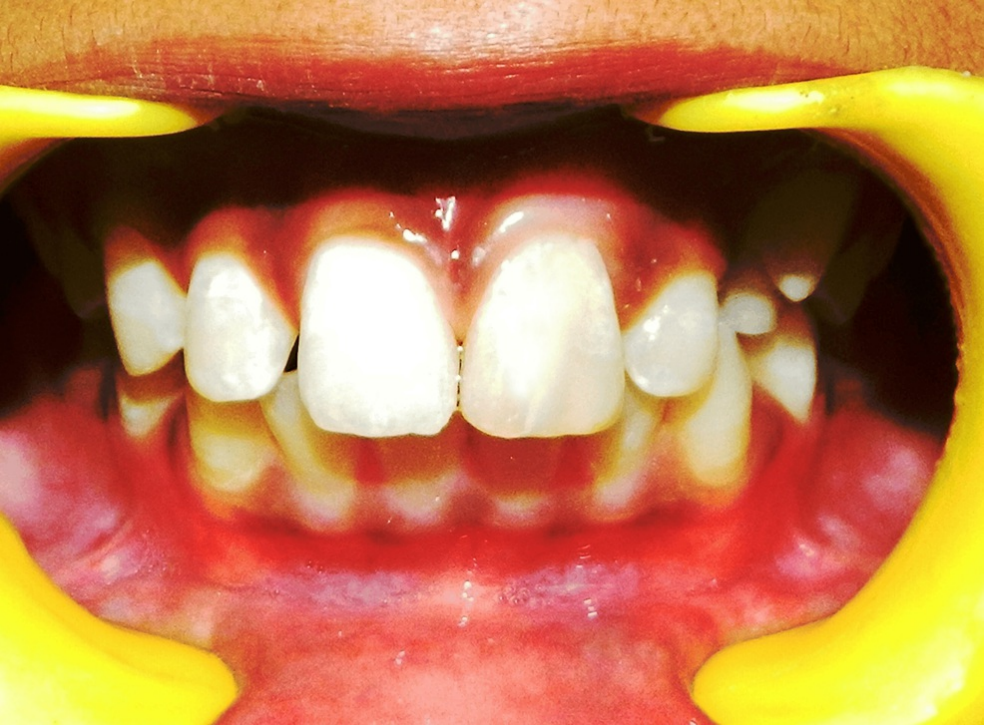
Post-operative intraoral photograph showing esthetically rehabilitated 21

The intracanal medicament was flushed out from the canal, copious irrigation was carried out, the canal was dried, and the tooth was then obturated with gutta-percha using lateral condensation technique with controlled pressure, followed by restoration with composite resin (Filtek Z350, 3M ESPE, St. Paul, MN, USA) (Figure 6).

**Figure 7:**
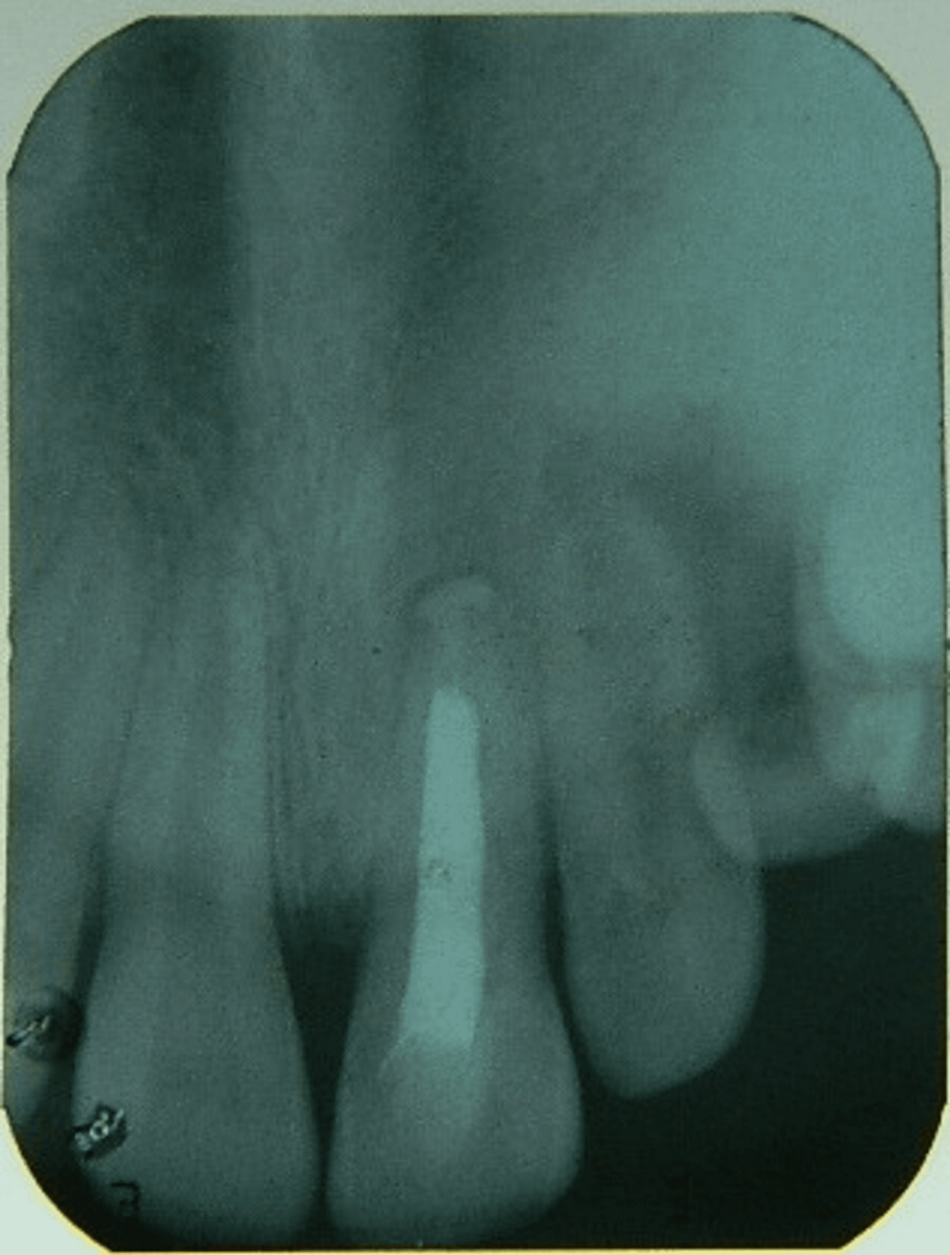
Post-operative IOPA (12 months follow-up) showing partial obliteration of the periodontal space IOPA: intraoral periapical

At the six-week follow-up visit, clinical examination showed normal probing sulcular depth and normal gingiva. There were no signs of inflammation/infection, tenderness, pain, or discomfort. Hence, the Ribbond splint was removed. On vertical percussion, a dull sound was observed, and partial periodontal space obliteration was noticed, on the distal aspect of the root. No pathological findings were observed after one year of follow-up (Figure 7).

## Discussion

According to dental traumatology literature, crown-root fractures are among the most difficult fracture types. The primary goal of treatment of the tooth affected with crown-root fracture is to restore the esthetic and function. Successful treatment results when a case is appropriately chosen based on clinical and radiographic examinations. Of crown-root fractures, VRFs account for 2-5%, with endodontically treated teeth having the highest incidence of 3.69-25% [5]. Vertical fractures are most commonly seen in the buccolingual direction due to greater mineralization and the dentin adaptation to functional stress-strain [6]. Grippo claimed that a variety of mechanical loading factors, including force magnitude, frequency, direction, location, and duration, have an impact on the teeth. Because of this, the stress placed on the tooth structure can lead to various fracture patterns, depending on the biological or anatomical characteristics of the root, crown, or supporting bone [7]. True VRFs in non-endodontically treated teeth have been documented previously by Liao et al. [8], Yeh [9], and Chan et al. [10]. The specific cause of these fractures is still unclear. External root resorption and failure often results from root surface drying during extraoral manipulations; thus, the tooth was held in wet betadine gauze. Literature suggests various time intervals for maintaining the intracanal calcium hydroxide dressing, which can vary from three months to a year based on the severity of the injury [11,12]. In the present case, the calcium hydroxide dressing was regularly changed and maintained for four weeks. Splinting is required after replantation to limit tooth mobility and accelerate healing of the periodontal ligament. Thus, after replantation, a semi-rigid esthetically acceptable polyethylene fiber splint (Ribbond) was given so as to allow proper healing.

Intentionally replanted teeth have been reported to have a retention rate of about 52-95% [13]. In some case reports, clinicians have removed the fractured tooth segment and used biocompatible substances like cyanoacrylates, silver glass ionomer cement, calcium hydroxide, and mineral trioxide aggregate to bond the root. Bender and Rossman [14] examined 31 cases, yielding an 80.6% success rate overall. Aqrabawi [15] evaluated two cases of intentional replantation where there was no sign of pathologic resorption at the five-year follow-up visit. Peer [16] reviewed nine cases of intentional replantation that illustrated the viability of the procedure for a range of indications including vertical fracture cases. Since mild external root resorption may not be appreciated radiographically, the periodontal health of the tooth is a more reliable indicator of the prognosis. However, this is directly related to the extraoral period of the tooth during the procedure. As the entire procedure took approximately nine minutes to complete, this may have reasonably contributed to the good prognosis of this case. As Grossman put it, intentional replantation should no longer be viewed as a "last resort" procedure reserved for hopeless teeth [17] and certainly not as Weine perceived it, "a procedure with the poorest prognosis" [18]. Although the success rate is not always high, intentional replantation is a treatment option for vertical crown-root fractures in order to preserve the natural dentition and prevent tooth extraction. 

## Conclusions

Intentional replantation was a successful management strategy for the clinical case described in this article. As it is a reliable and predictable procedure, it should be more frequently taken into consideration as a viable treatment option to preserve the natural dentition. 

## References

[1] Panangipalli SS, Vasepalli M, Punithavathy R, Martha S, Birapu UC, Raparla M (2022). Prevalence of traumatic injuries to permanent anterior teeth and predisposing risk factors among government and private school children of Kakinada and Rajanagaram of East Godavari District. Int J Clin Pediatr Dent.

[2] Zhou ZL, Gao L, Sun SK (2022). Spontaneous healing of complicated crown-root fractures in children: two case reports. World J Clin Cases.

[3] Sanaei‐rad P, Hajihassani N, Jamshidi D (2020). Management of a complex traumatic dental injury: crown, crown‐root, and root fracture. Clin Case Rep.

[4] Asgary S (2011). Management of a hopeless mandibular molar: a case report. Iran Endod J.

[5] Cohen S, Blanco L, Berman L (2003). Vertical root fractures: clinical and radiographic diagnosis. J Am Dent Assoc.

[6] Kishen A, Kumar GV, Chen NN (2004). Stress-strain response in human dentine: rethinking fracture predilection in postcore restored teeth. Dent Traumatol.

[7] Grippo JO (1991). Abfractions: a new classification of hard tissue lesions of teeth. J Esthet Dent.

[8] Liao WC, Chen CH, Pan YH, Chang MC, Jeng JH (2021). Vertical root fracture in non-endodontically and endodontically treated teeth: current understanding and future challenge. J Pers Med.

[9] Yeh CJ (1997). Fatigue root fracture: a spontaneous root fracture in non-endodontically treated teeth. Br Dent J.

[10] Chan CP, Tseng SC, Lin CP, Huang CC, Tsai TP, Chen CC (1998). Vertical root fracture in nonendodontically treated teeth--a clinical report of 64 cases in Chinese patients. J Endod.

[11] Hosoya N, Takahashi G, Arai T, Nakamura J (2001). Calcium concentration and pH of the periapical environment after applying calcium hydroxide into root canals in vitro. J Endod.

[12] Estrela C, Sydney GB, Pesce HF, Felippe Júnior O (1995). Dentinal diffusion of hydroxyl ions of various calcium hydroxide pastes. Braz Dent J.

[13] Andreasen JO, Borum MK, Jacobsen HL, Andreasen FM (1995). Replantation of 400 avulsed permanent incisors. 4. Factors related to periodontal ligament healing. Endod Dent Traumatol.

[14] Bender IB, Rossman LE (1993). Intentional replantation of endodontically treated teeth. Oral Surg Oral Med Oral Pathol.

[15] Aqrabawi J (1999). Five-year follow-up of successful intentional replantation. Dent Update.

[16] Peer M (2004). Intentional replantation - a 'last resort' treatment or a conventional treatment procedure? nine case reports. Dent Traumatol.

[17] Grossman LI (1966). Intentional replantation of teeth. J Am Dent Assoc.

[18] Weine FS (1980). The case against intentional replantation. J Am Dent Assoc.

